# Association of dietary niacin intake with the prevalence and incidence of chronic obstructive pulmonary disease

**DOI:** 10.1038/s41598-024-53387-4

**Published:** 2024-02-04

**Authors:** Wen-Wen Li, Kun-Lun Ren, Jia Yu, Hai-Sheng Guo, Ben-Hong Liu, Yang Sun

**Affiliations:** Dongying People’s Hospital, Shandong, Dongying China

**Keywords:** Diseases, Medical research, Risk factors

## Abstract

Evidence regarding the association between dietary niacin intake and chronic obstructive pulmonary disease (COPD) is limited. Our study investigates the relationship between dietary niacin intake and the prevalance and incidence of COPD in the adult population of the United States, using data from the National Health and Nutrition Examination Survey (NHANES) from 2003 to 2018. Data on niacin intake were extracted through dietary intake interviews. COPD diagnoses were based on lung function, medical history, and medication usage. We analyzed the association between niacin consumption and COPD using multiple logistic regression and restricted cubic spline models. The study included 7055 adult participants, divided into COPD (n = 243; 3.44%) and non-COPD (n = 6812; 96.56%) groups. Those with COPD had lower average niacin intake (21.39 ± 0.62 mg/day) compared to the non-COPD group (25.29 ± 0.23 mg/day, p < 0.001). In the adjusted multivariable model, the odds ratios (OR) and 95% confidence intervals (CI) for COPD in the highest versus lowest quartile of dietary niacin intake were 0.55 (0.33 to 0.89, P for trend = 0.009). Subgroup analysis, after adjustment for various variables, revealed no significant interaction effects. Dietary niacin intake was inversely associated with COPD prevalence in US adults. Participants with the highest dietary niacin intake demonstrated the lowest odds of COPD. The potential of dietary niacin supplementation as a strategy to mitigate COPD warrants further investigation.

## Introduction

Chronic obstructive pulmonary disease (COPD) is a prevalent lung condition, primarily characterized by airflow obstruction resulting from abnormalities in the airways and alveoli^1^. The primary clinical symptoms of COPD include dyspnea, cough, and sputum production. Importantly, the impact of COPD extends beyond the lungs, affecting the bones, muscles, and cardiovascular system. Currently, COPD is responsible for over three million deaths annually worldwide, significantly burdening global public health. Despite notable advancements in treatment methods and medical equipment, the mortality rate associated with COPD remains high. Factors such as the increasing prevalence of smoking and aging contribute to a projection that COPD will result in more than 5.4 million deaths annually by 2060^1^. Consequently, identifying and addressing modifiable risk factors is essential in preventing or delaying the onset of COPD and mitigating its complications.

Vitamins are essential nutrients that play a crucial role in maintaining normal physiological functions in humans and animals. Deficiencies in these nutrients can lead to various health issues, while their replenishment often alleviates related deficiency symptoms. Research has demonstrated that certain vitamins, including vitamin C, vitamin E, vitamin D, and niacin, are effective in reducing oxidative stress. This reduction is achieved through the decrease of lipid peroxidation, protein carbonylation, and advanced glycated end-products^2^. Furthermore, vitamins with antioxidant properties contribute to cellular repair and inflammation reduction. For instance, a Korean study on the intake of antioxidant vitamins and lung function highlighted the positive impact of these vitamins on lung health^3^. More recent research has established a link between vitamin supplementation and a decreased risk of developing asthma and other respiratory conditions^4,5^.

Niacin, a member of the B vitamin group, is also recognized as nicotinic acid or vitamin B3. Research has shown that certain vitamins, notably vitamins A, C, D, and E, have a positive impact on COPD^6–8^. These vitamins can be ingested either as supplements or through a well-balanced diet. However, the relationship between vitamin B3 and COPD remains unexplored. Thus, this study is focused on examining the link between COPD prevalence and dietary intake of niacin.

## Methods

### Data source

**Figure 1:**
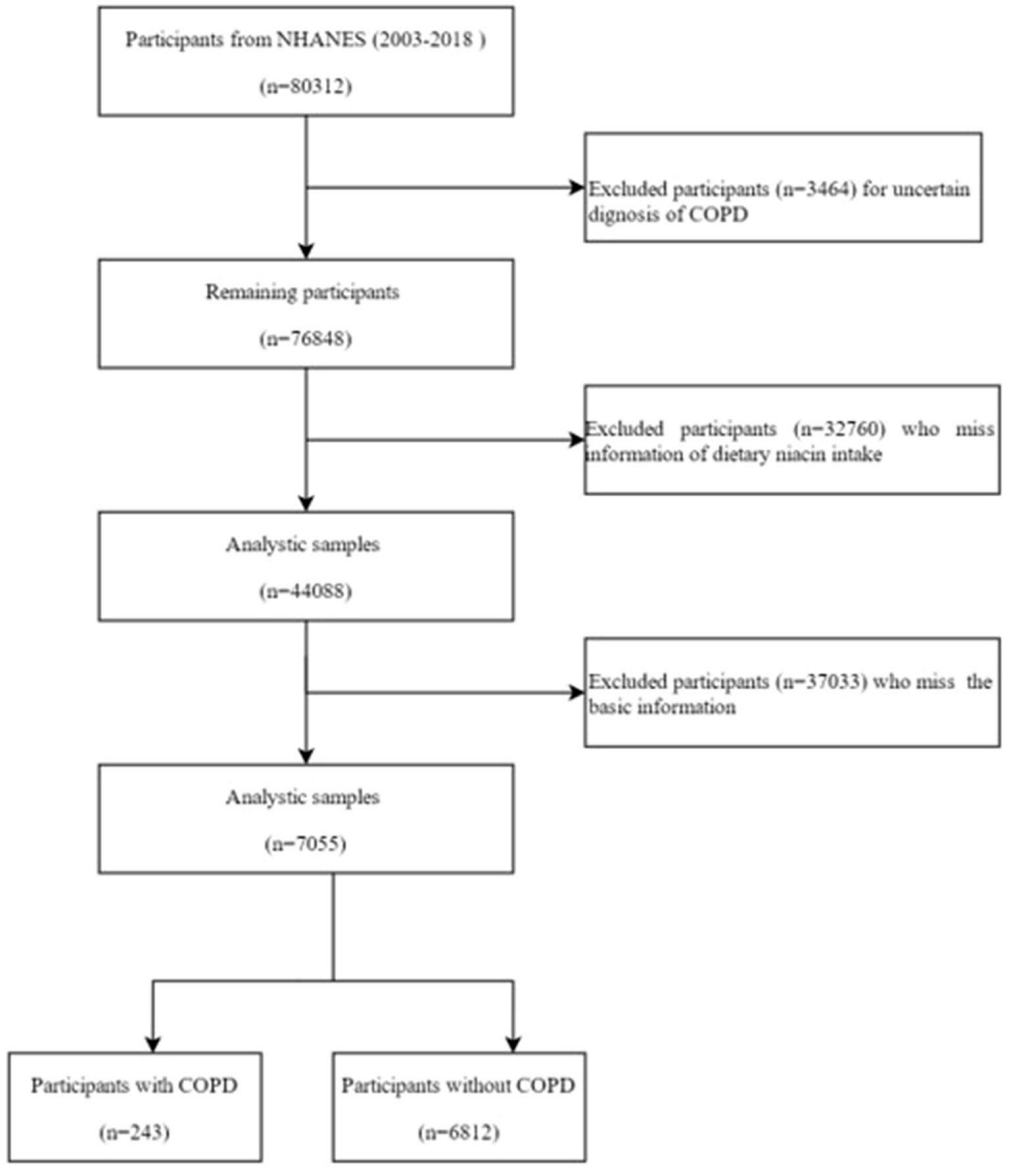
Flow chart of the screening process for the selection of eligible participants. NHANES, National Health and Nutrition Examination Survey.

The National Health and Nutrition Examination Survey (NHANES) is a program initiated by the U.S. government to assess the health and nutritional status of Americans. It comprises a series of interviews conducted in participants’ homes, coupled with health examinations in mobile examination centers. The survey gathers a wide array of data, including demographic, socioeconomic, dietary, and health information, through questionnaires and physical and laboratory examinations^9^. The NHANES database, updated biennially, is globally accessible and contains comprehensive datasets. For this study, data from 2003 to 2018 were retrieved, focusing on individuals with dietary niacin intake and COPD. Initially, the dataset included 80,312 participants, but exclusions were made for incomplete data. Participants with missing COPD information (3464), lacking dietary niacin intake data (32,760), or without basic demographic information (37,033) were removed from the analysis. Ultimately, 7055 participants were included in the study (Fig. 1). The research protocol was approved by the National Center for Health Statistics (NCHS) Research Ethics Review Board, and all participants provided written informed consent^10^. The NHANES employs a stratified, multi-stage probability sampling design, ensuring that the sample is representative of the civilian, non-institutionalized U.S. population.

### Dietary niacin intake assessment

The NHANES employs the U.S. Department of Agriculture’s (USDA) dietary data collection tool, which uses the automated multiple pass method, to gather two 24-h dietary recalls from participants. The first recall is conducted in-person at a mobile examination center, while the second occurs via telephone interview, generally 3 to 10 days later. To calculate nutrient intakes, the USDA's Food and Nutrient Database for Dietary Studies (FNDDS version 2.0), is utilized, the detailed instructions as showed in the appendix^11^. For the purpose of our study, only the first 24-h dietary recall data was used to represent dietary intake, ensuring higher response rates and accuracy. Using this method, daily dietary niacin, energy, alcohol, and macronutrient intakes were determined for each survey cycle.

### Outcome ascertainment

The primary event of interest is the occurrence of COPD. COPD diagnosis was determined based on self-reported physician diagnosis, aligning with methodologies used in previous studies^12^. Specifically, a diagnosis of COPD was identified if participants affirmatively responded to any of the following questions: "Has a doctor or other health professional ever told you that you had COPD?", "Has a doctor or other health professional ever told you that you had chronic obstructive pulmonary disease or COPD?", or "Have you been diagnosed with emphysema or chronic bronchitis?". Individuals who answered "yes" to any of these questions were categorized into the COPD group, while those who answered "no" were placed in the non-COPD group. Additionally, participants were included in the COPD group if they had pulmonary function test results with a forced expiratory volume in 1 s (FEV_1_) to forced vital capacity (FVC) ratio below 0.7. The COPD group also encompassed individuals over 40 years old with a history of smoking or chronic bronchitis who were using specific medications, including selective phosphodiesterase-4 inhibitors, mast cell stabilizers, leukotriene modifiers, and inhaled corticosteroids.

### Covariates

In this study, demographic data such as gender, age, race, family income, education level, smoking status, marital status, drinking habits, and histories of diabetes and hypertension were gathered through household interviews using standardized questionnaires. Physical data, including body weight, height, and blood pressure, were collected during examinations at the Mobile Examination Center. Additionally, participants provided blood samples for baseline plasma glucose measurements. Body mass index (BMI) was calculated using weight in kilograms divided by the square of height in meters. Race was categorized into Mexican American, non-Hispanic White, other Hispanic, non-Hispanic Black, and other races. Education levels were classified into three groups: those who did not complete high school, those who completed high school or equivalent, and those with higher education. Annual family income was segmented into below and above $20,000. Smoking status was divided into three categories: never smoked or less than 100 cigarettes in lifetime, current smokers, and former smokers. Marital status was classified as married/living with a partner, never married, or widowed/divorced/separated. Drinking status was categorized into former, heavy, mild, moderate, and never drinkers. Diabetes was identified by one or more of the following criteria: a fasting blood glucose level ≥ 7.0 mmol/L, a 2-h plasma glucose level ≥ 11.1 mmol/L, a self-reported history of diabetes, or the use of diabetes medications. Hypertension was defined as having a systolic blood pressure over 130 mmHg, a diastolic blood pressure over 80 mmHg, using hypertension medication, or a self-reported history of hypertension. For an in-depth explanation of covariate adjustments, refer to previously published studies^13–15^.

### Statistical analysis

The NHANES database employs a complex, multi-stage sampling method. Accordingly, we used the weights in accordance with dietary interview (WTDRD1 and WTDRD2) for analysis. Dietary niacin intake was categorized into quartiles and treated as a categorical variable. We reported continuous data as either mean with standard deviation or median with interquartile range, and categorical data as counts and percentages. To compare continuous variables and constituent ratios across groups, we employed various statistical tests. The Student’s *t*-test or one-way Analysis of variance (ANOVA) was used for continuous variables, and the chi-square test or Fisher's exact test for constituent ratios. To explore the relationship between dietary niacin intake and COPD prevalence, logistic regression analysis was conducted. We established three models: Model 1, unadjusted; Model 2, adjusted for age; and Model 3, adjusted for age, gender, race, BMI, education level, annual household income, smoking status, drinking status, marital status, hypertension, and diabetes.

To address the non-linear relationships between continuous variables and response variables, our study employed restricted cubic splines. These are particularly effective in identifying critical points in such relationships^16,17^. We used these splines to explore the non-linear relationship between niacin intake and the prevalence of COPD, while controlling for other variables. Additionally, subgroup analyses for each confounding variable listed in the baseline table were conducted using hierarchical logistic regression models. All statistical tests in this study were two-sided, with a P-value < 0.05 considered statistically significant. The analysis was conducted using R software (version 4.2.2).

### Ethics approval and consent to participate

The National Center for Health Statistics Institutional Review Board examined and authorized the studies that involved human subjects. All methods were performed in accordance with the relevant guidelines and regulations. In order to take part in this study, the patients/participants gave their written informed consent.

## Results

### Baseline characteristics

From the 2003–2018 NHANES dataset, 80,312 participants were initially considered. However, after excluding those with incomplete data on COPD, diabetes, and hypertension, as well as other essential information relevant to this study, the final sample size was reduced to 7055 participants. These participants were divided into two groups: the COPD group (n = 243) and the non-COPD group (n = 6812) (Fig. 1). Table 1 presents the characteristics of these groups. The percentage of people with COPD was 3.44%. Compared to the non-COPD group, individuals with COPD were older (60.75 ± 1.10 years vs. 46.69 ± 0.49 years, p < 0.001) and had a lower average daily niacin intake (21.39 ± 0.62 mg/day vs. 25.29 ± 0.23 mg/day, p < 0.001). Additionally, the COPD group tended to come from families with lower annual incomes and showed differences in racial composition and marital status compared to the non-COPD group. As anticipated, the incidence of smoking, alcohol consumption, diabetes, and hypertension was higher in the COPD group than in the non-COPD group.

**Table 1:** The characteristic of included subjects between different ePVS levels.

Variable	Total (n = 7055)	Non-COPD (n = 6812)	COPD (n = 243)	P value
Niacin intake (mg/day)	25.17 ± 0.23	25.29 ± 0.23	21.39 ± 0.62	< 0.001
Gender				0.080
Man	3561 (50.47%)	3427 (48.69%)	134 (43.27%)	
Female	3494 (49.53%)	3385 (51.31%)	109 (56.73%)	
Age (year)				< 0.001
< 30	1127 (15.97%)	1126 (17.49%)	1 (0.57%)	
30–50	2475 (35.08%)	2438 (43.87%)	37 (23.91%)	
> 50	3453 (48.94%)	3248 (38.63%)	205 (75.52%)	
Mean ± SD	47.13 ± 0.49	46.69 ± 0.49	60.75 ± 1.10	< 0.001
BMI (kg/m^2^)				0.730
< 25	1628 (23.08%)	1568 (25.18%)	66 (25.98%)	
25–30	2052 (29.09%)	60 (27.88%)	3258 (46.41%)	
> 30	3375 (47.84%)	1986 (28.41%)	117 (46.14%)	
Mean ± SD	28.52 ± 0.16	28.51 ± 0.17	28.67 ± 0.73	0.830
Race				< 0.001
Mexican American	1323 (18.75%)	1311 (7.27%)	12 (0.74%)	
Non-hispanic black	1429 (20.26%)	1393 (10.33%)	36 (5.94%)	
Non-hispanic white	3833 (54.33%)	3648 (74.05%)	185 (88.32%)	
Other hispanic	211 (2.99%)	210 (3.51%)	1 (0.21%)	
Other race	259 (3.67%)	250 (4.84%)	9 (4.79%)	
Income				0.02
< 20,000	1529 (21.67%)	1459 (15.16%)	70 (22.84%)	
≥ 20,000	5526 (78.33%)	5353 (84.84%)	173 (77.16%)	
Education				0.09
<High school	1872 (26.53%)	1792 (15.85%)	80 (23.95%)	
=High school	1739 (24.65%)	1683 (25.83%)	56 (24.48%)	
>High school	1739 (24.65%)	3337 (58.32%)	107 (51.57%)	
Smoking				< 0.001
Former	1950 (27.64%)	1813 (25.06%)	137 (53.78%)	
Never	3559 (50.45%)	3527 (51.44%)	32 (13.74%)	
Now	1546 (21.91%)	1472 (23.50%)	74 (32.48%)	
Alcohol				< 0.001
Former	1577 (22.35%)	1474 (17.61%)	103 (35.45)	
Heavy	1298 (18.4%)	1266 (20.41%)	32 (16.53%)	
Mild	2239 (31.74%)	2169 (34.21%)	70 (29.72%)	
Moderate	1000 (14.17%)	977 (16.51%)	23 (12.54%)	
Never	941 (13.34%)	926 (11.28%)	15 (5.76%)	
Hypertension				< 0.001
No	3933 (55.75%)	3866 (62.80%)	67 (32.28%)	
Yes	3122 (44.25%)	2946 (37.20%)	2946 (37.20%)	
Diabetes				< 0.001
No	1076 (15.25%)	1019 (11.01%)	57 (20.13%)	
Yes	5979 (84.75%)	5793 (88.99%)	186 (79.87%)	
Marital status				< 0.001
Married/living with partner	4401 (62.38%)	4255 (66.36%)	146 (66.35%)	
Never married	1052 (14.91%)	1036 (15.27%)	16 (5.97%)	
Widowed/divorced/separated	1602 (22.71%)	1521 (18.36%)	81 (27.67%)	

Data are presented as N% (χ^2^-test) or Mean ± SD (independent t-test).

BMI, body mass index.

^a^Weighted comparison.

### Multivariate regression analysis

The multivariate regression analyses, adjusted for various confounding variables, consistently revealed a negative correlation between dietary niacin intake and the prevalence of COPD across all three models. Specifically, participants in the highest quartile of niacin intake (Q4, 29.388–122.087 mg/day) showed a significantly lower risk of COPD compared to those in the lowest quartile (Q1). The odds ratios were 0.41 (0.28–0.60) in Model 1, 0.56 (0.37–0.85) in Model 2, and 0.55 (0.33–0.89) in Model 3. Notably, the trend across all three models was statistically significant, with P-values for the trend being less than 0.05 (Table 2).

**Table 2:** ORs and 95% CIs for COPD according quartile of niacin intake.

Variables	OR (95%CI)					
	Model 1	P-value	Model 2	P-value	Model 3	P-value
Niacin intake (mg/day)						
Q1 (1.771, 16.074)	1 (ref)		1 (ref)		1 (ref)	
Q2 (16.075, 21.895)	0.82 (0.53, 1.28)	0.371	0.82 (0.53, 1.29)	0.377	0.81 (0.50, 1.316)	0.354
Q3 (21.896, 29.387)	0.64 (0.42, 0.96)	0.034	0.70 (0.46, 1.06)	0.091	0.66 (0.42, 1.04)	0.067
Q4 (29.388, 122.087)	0.41 (0.28, 0.60)	< 0.001	0.56 (0.37, 0.85)	0.008	0.55 (0.33, 0.89)	0.021
P for trend	< 0.0001		0.004		0.009	

OR, odds ratio; CI, confidence interval; Q, quartile. Model 1 is unadjusted. Model 2 adjusted for age. Model 3 adjusted for age, race, annual family income, smoking status, hypertension, diabetes, marital status, drinking status. The lowest quartile of dietary niacin intake was used as the reference group.

### Nonlinear analysis

**Figure 2:**
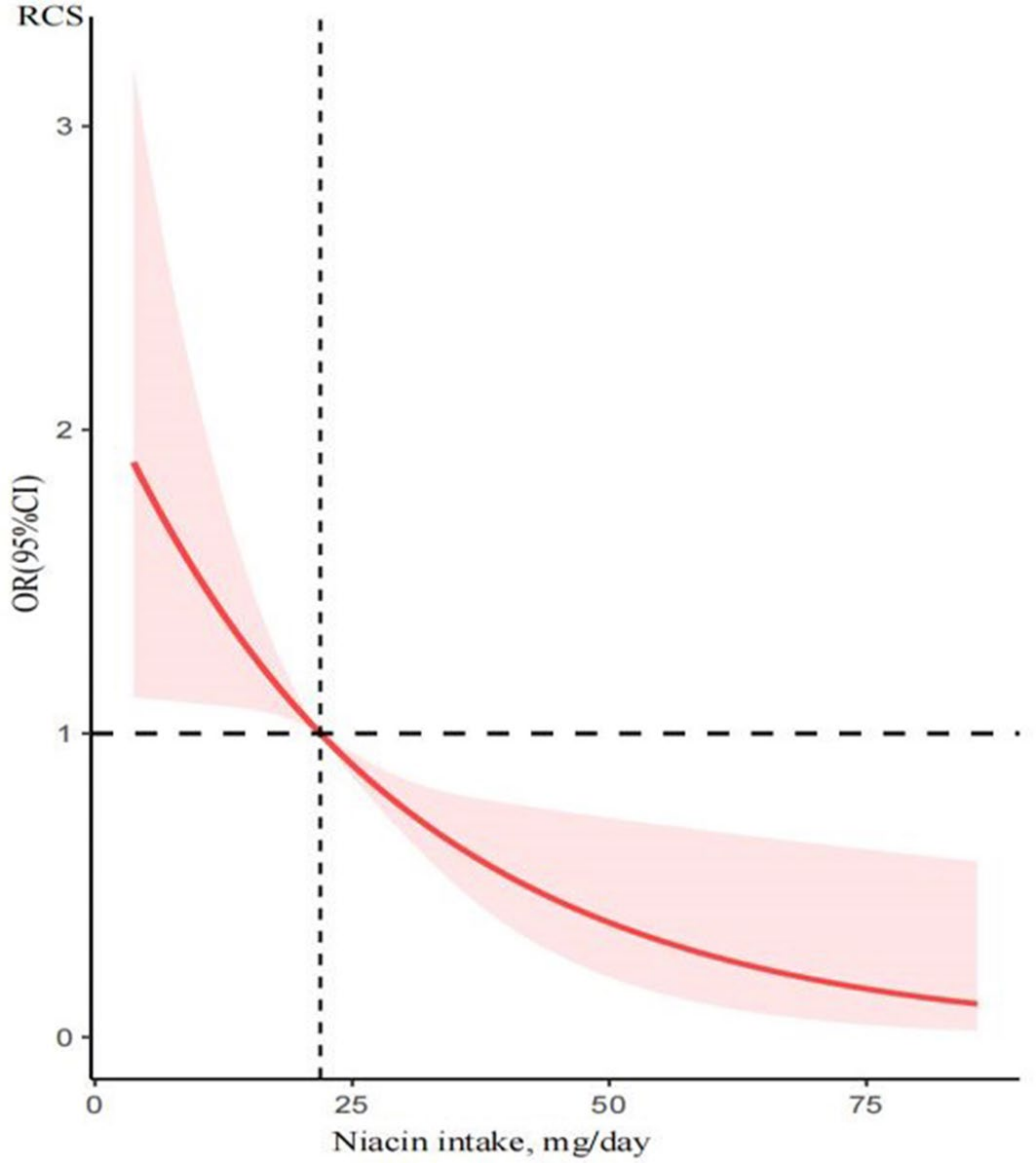
The restricted cubic spline model showed a dose–response relationship between dietary niacin intake and COPD. P for non-linearity = 0.98.

We identified a clear dose–response relationship between dietary niacin intake and COPD prevalence (Fig. 2). The analysis revealed a linear association between these two variables, evidenced by a P value of 0.98 for non-linearity. This suggests a consistent decrease in COPD prevalence with increasing levels of dietary niacin intake.

### Subgroup or interaction analyses

**Figure 3:**
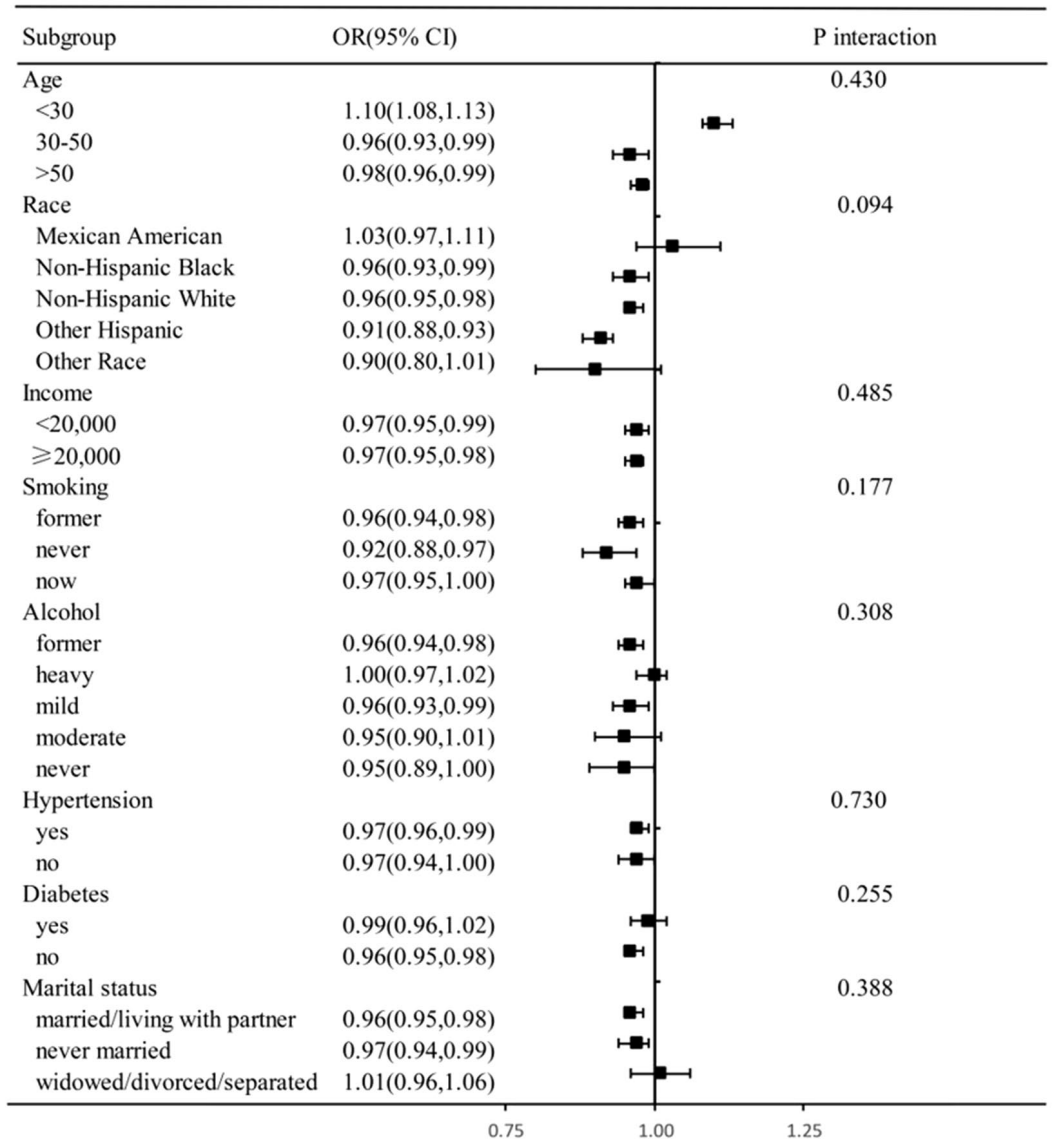
The subgroup and interaction analyses for dietary niacin intake on COPD. Stratified logistic regression analysis to identify variables that modify the correlation between dietary niacin intake and COPD. A subgroup and interaction analyses with dietary niacin intake as a categorical variable (divided into quarters), compared Q4 with Q1. Adjusted for age, race, annual family income, smoking status, hypertension, diabetes, marital status, drinking status.

In our analysis, we applied Model 3, which includes multiple variables, to various subgroups. These subgroups were defined based on different levels of dietary niacin intake, categorized into four distinct quartiles. After adjusting for the factors included in Model 3, the subgroup analysis revealed a consistent relationship between dietary niacin intake and COPD prevalence across different demographics and health-related factors. These factors included age, race, income, drinking habits, smoking status, hypertension, diabetes, and marital status (Fig. 3). In the subgroup analysis, we found the protective effect of dietary niacin intake on COPD was independent of income, smoking, hypertension, diabetes. There's no evidence suggested age (P interaction = 0.430), race (P interaction = 0.094), income (P interaction = 0.485), drinking habits (P interaction = 0.308), smoking status (P interaction = 0.177), hypertension (P interaction = 0.730), diabetes (P interaction = 0.255), and marital status (P interaction = 0.388) have no interact effect on the protective effect of dietary niacin intake on COPD.

## Discussion

Our study established a distinct correlation between niacin intake and the prevalence of COPD in adults. The dose–response analysis demonstrated a linear relationship between the level of niacin consumption and the incidence of COPD. We observed that within a suitable range, higher niacin intake is associated with a decreased likelihood of developing COPD in adults. These findings suggest that niacin could serve as a protective factor against the onset of COPD.

Diet plays a crucial role in managing and preventing various chronic diseases, including cardiovascular diseases, cancer, diabetes, and lung diseases, which collectively account for over 70% of deaths^18–20^. Niacin, a vital nutrient, is essential for metabolism, DNA repair, and nervous system function^21^. It is available as a supplement, commonly used in high doses to treat conditions like hyperlipidemia and pellagra^22,23^. Furthermore, niacin supplementation has been shown to positively impact cancer mortality. A cohort study with 3504 participants demonstrated a negative association between niacin intake and cancer mortality, with significant P values in all models^24^. This correlation persisted across various subgroups, including sex, age, and BMI. Kaplan–Meier survival analyses revealed higher survival rates in groups with high niacin intake compared to those with low intake, although this did not extend to all-cause mortality^24^. In the context of lung health, niacin has demonstrated benefits in reducing inflammation. Research indicates that high doses of niacin can lessen lung inflammation in rats by modulating the NF-κB pathway^25^. This includes reducing pro-inflammatory cytokines, mitigating histologic lung damage, and improving survival rates during sepsis^25^. Additionally, a balanced diet contributes to reducing airway irritation and enhancing energy production. A study by Taeyun et al., based on the Korea National Health and Nutrition Examination Survey and involving 22,948 participants, found that niacin, along with other nutrients like vitamin A, fiber, carbohydrates, protein, riboflavin, and vitamin C, was associated with decreased severity of airway damage in COPD patients^26^.

Niacin, found in foods like meat, fish, and nuts, plays a significant role in preventing and managing COPD. Numerous studies highlight the importance of nutrition in both averting and treating COPD. For instance, recent large-scale studies in China have established a correlation between regular fish consumption, as part of a healthy lifestyle, and a reduced risk of COPD, likely due to fish's niacin content^27^. COPD patients often experience pulmonary arterial constriction and spasms due to restricted airflow and chronic hypoxia, leading to pulmonary hypertension, a common COPD complication^28,29^. Niacin shows promising effects in mitigating pulmonary arterial hypertension (PAH). It has been observed to slow the progression of PAH by reducing vascular remodeling and promoting the release of prostaglandin D2 (PGD2) from macrophages^30^. A nutrigenomics study revealed an association between niacin intake and improved FEV_1_ in chronic smokers^31^.

The mechanisms by which dietary niacin intake impacts COPD remain to be fully elucidated. COPD, a progressive lung disease, is characterized by inflammation and oxidative stress^32^. Mitigating oxidative stress involves bolstering antioxidant capacity through both endogenous antioxidants (like superoxide dismutase [SOD], catalase [CAT], and glutathione reductase [GR]) and exogenous sources such as dietary antioxidants, including tocopherols, ascorbic acid, carotenoids, niacin, and certain trace elements. Research indicates that niacin and its primary metabolite have antioxidant and anti-inflammatory effects. These effects are manifested in the attenuation of reactive oxygen species (ROS), nitric oxide (NO), and pro-inflammatory cytokine production in activated human mature macrophages^33^. Given these properties, niacin is likely to aid in preventing COPD by reducing inflammation in the airways, protecting them from oxidative damage, and improving their overall functionality.

Our study boasts several strengths, including a comprehensive approach with a large and diverse sample encompassing various racial, social, and geographical backgrounds. This diversity allowed us to explore the relationship between dietary niacin intake and COPD. One of the key strengths is that our subjects were representative of the general U.S. population with COPD, enhancing the generalizability of our findings.

However, there are limitations need to be addressed. One potential issue is confounding by unmeasured factors, such as lifestyle choices, which may influence dietary habits. Additionally, the cross-sectional design precludes establishing a cause-and-effect relationship between niacin intake and COPD. To confirm niacin's benefits in COPD, further research, such as controlled trials or longitudinal epidemiological studies, is needed. Another limitation is the reliance on a food frequency questionnaire for dietary intake data, which may be subject to recall and misclassification biases. However, these biases likely do not differentially affect the COPD and non-COPD groups. Also, the variability in niacin bioavailability among participants could lead to inaccuracies in assessing its dietary effects. In addition, in nutrition research when two 24-hour recalls are collected, typically the two recalls are averaged together for several reasons including increased precision, reduction of memory bias, and to include variability of food consumption during the week, to identify outliers among others. We only used one 24 h recall data in this study which may has some effect on the stability of our results. Future research might benefit from serum-based analyses to more accurately determine the direct relationship between niacin levels and COPD. In conclusion, while our study sheds light on the potential role of niacin in COPD, further investigation is necessary. This could include cohort studies or randomized controlled trials (RCTs) focusing on the preventive or therapeutic effects of niacin on COPD.

## Conclusion

Our study found that higher dietary niacin intake correlates with a lower prevalence of COPD in adults, with this protective effect intensifying as niacin consumption increases. This observation offers preliminary evidence of a potential link between niacin intake and reduced COPD incidence. However, further research is required to ascertain whether niacin as an alternative therapy can enhance lung ventilation function and effectively prevent COPD. Additionally, there is a need for more detailed studies to establish the optimal dosage and timing for niacin's potential beneficial effects on COPD (Supplementary Information).

### Supplementary Information

Supplementary Information 1.

## Data Availability

The data analyzed in this study is accessible via the internet. The details of the repository/repositories, including the accession number, are available here: http://www.cdc.gov/nchs/nhanes.htm.
