# Supplementary figures and images for: Association of dietary niacin intake with the prevalence and incidence of chronic obstructive pulmonary disease
Source: Sci Rep. 2024 Feb 4;14:2863. doi: 10.1038/s41598-024-53387-4 (PMC10838909; doi:10.1038/s41598-024-53387-4)

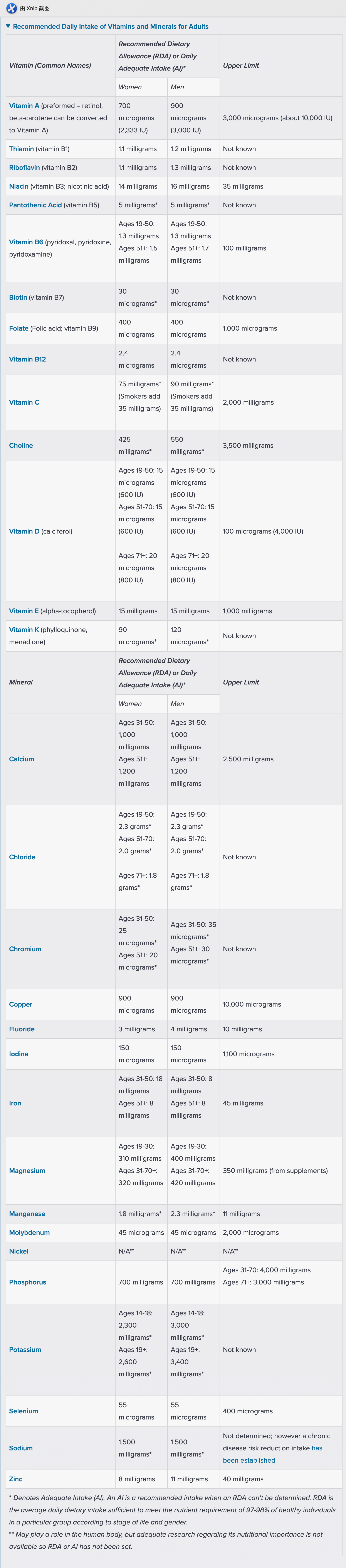

Supplement: Supplementary file 1 — Supplementary Information 1. [file 41598_2024_53387_MOESM1_ESM.png]
